# A 6 Week Randomized Double-Blind Placebo-Controlled Trial of Ziprasidone for the Acute Depressive Mixed State

**DOI:** 10.1371/journal.pone.0034757

**Published:** 2012-04-24

**Authors:** Ashwin Patkar, William Gilmer, Chi-un Pae, Paul A. Vöhringer, Michael Ziffra, Edward Pirok, Molly Mulligan, Megan M. Filkowski, Elizabeth A. Whitham, Niki S. Holtzman, Sairah B. Thommi, Tanya Logvinenko, Antony Loebel, Prakash Masand, S. Nassir Ghaemi

**Affiliations:** 1 Department of Psychiatry, Duke University Medical Center, Durham, North Carolina, United States of America; 2 Department of Psychiatry, Northwestern University, School of Medicine, Chicago, Illinois, United States of America; 3 Mood Disorders Program, Department of Psychiatry, Tufts Medical Center, Boston, Massachusetts, United States of America; 4 Hospital Clinico, Facultad de Medicina, Universidad Chile, Santiago, Chile; 5 Department of Psychiatry, Emory University School of Medicine, Atlanta, Georgia, United States of America; 6 Biostatistics Research Center at the Institute for Clinical Research and Health Policy Studies, Tufts Medical Center, Boston, Massachusetts, United States of America; 7 Sunovion Pharmaceuticals, Fort Lee, New Jersey, United States of America; 8 Tufts University School of Medicine, Boston, Massachusetts, United States of America; 9 Graduate Program, Clinical and Translational Science Institute, Sackler School of Graduate and Biomedical Sciences, Tufts University, Boston, Massachusetts, United States of America; Catholic University of Sacred Heart of Rome, Italy

## Abstract

**Objective:**

To examine the efficacy of ziprasidone vs. placebo for the depressive mixed state in patients with bipolar disorder type II or major depressive disorder (MDD).

**Methods:**

73 patients were randomized in a double-blinded, placebo-controlled study to ziprasidone (40-160 mg/d) or placebo for 6 weeks. They met DSM-IV criteria for a major depressive episode (MDE), while also meeting 2 or 3 (but not more nor less) DSM-IV manic criteria. They did not meet DSM-IV criteria for a mixed or manic episode. Baseline psychotropic drugs were continued unchanged. The primary endpoint measured was Montgomery- Åsberg Depression Rating Scale (MADRS) scores over time. The mean dose of ziprasidone was 129.7±45.3 mg/day and 126.1±47.1 mg/day for placebo.

**Results:**

The primary outcome analysis indicated efficacy of ziprasidone versus placebo (p = 0.0038). Efficacy was more pronounced in type II bipolar disorder than in MDD (p = 0.036). Overall ziprasidone was well tolerated, without notable worsening of weight or extrapyramidal symptoms.

**Conclusions:**

There was a statistically significant benefit with ziprasidone versus placebo in this first RCT of any medication for the provisional diagnostic concept of the depressive mixed state.

**Trial Registration:**

Clinicaltrials.gov NCT00490542

## Introduction

Mixed states in bipolar disorder have long been recognized. Over a century ago, Weygandt argued that mixed states were the most common presentations in manic-depressive illness [1]. While Kraepelin identified pure depression and pure mania, he described six different mixed states, which he considered more prevalent than pure mood states [2].

In the Diagnostic and Statistical Manual of Mental Disorders (DSM-IV), the definition of a mixed episode was narrowed, compared to previous definitions, so as to require full criteria for both mania and depression. This was not primarily based on empirical grounds, but rather, according to some members of the DSM-IV committee [3], to compensate for expansion of the bipolar diagnosis into other areas (for example inclusion of type II and rapid cycling definitions).

In the last decade, a number of studies have suggested that the previous broader definitions of mixed states may have diagnostic validity and therapeutic utility. Empirical studies support the possible validity of mixed states in which manic episode criteria are met with two or more depressive criteria (dysphoric mania) [4]. Using this definition, for instance, one study demonstrated greater treatment response with valproate than with lithium [5]. Other clinical studies suggest that a mixed state may be present when major depressive episode (MDE) criteria are met with at least one manic criterion (such as racing thoughts) [6], [7]. In those clinical studies, such mixed states appeared less responsive to antidepressants and more responsive to adjunctive mood stabilizers [8].

Using the narrow DSM-IV criteria, less than 10% of episodes in patients with bipolar disorder meet criteria for a mixed episode. Using broader mixed states criteria, incorporating the clinical features of dysphoric mania and agitated depression, data suggest that about 50% of episodes in bipolar disorder would be diagnosable as mixed states [7].

One study found that only 54% of 143 broadly defined mixed states (using Kraepelinian definitions) met DSM-III-R criteria for a mixed episode [9]. The concept of the depressive mixed state is thus provisional, and not part of DSM definitions.

It should be noted that this study of the depressive mixed state differs from a study of “agitated depression.” The concept of agitated depression does not have a consensus definition; usually it entails a MDE with at least psychomotor agitation. Our definition of depressive mixed state is more than simply psychomotor agitation along with depression: there must also be one or two other manic symptoms (depending on the definition). Three or more manic symptoms would define the condition as a DSM-IV mixed episode. Thus the depressive mixed state is a subthreshold DSM-IV mixed episode. Studies suggest that the majority of persons with a depressive mixed state have bipolar disorder type II, but a substantial group will also have MDD, according to DSM-IV criteria [7].

The purpose of this study was to examine the efficacy of ziprasidone for the depressive mixed state in patients with bipolar or unipolar depression. This was the first double-blind randomized clinical trial of any medication in the depressive mixed state.

## Methods

The protocol for this trial and supporting CONSORT checklist are available as supporting information; see Checklist S1 and Protocol S1. This five-site, block randomized, double-blinded, placebo-controlled study analyzed 73 patients who were randomized to ziprasidone or placebo for 6 weeks between November 2006 and September 2009. The study was approved by the Institutional Review Boards at each participating site (Tufts Medical Center, Cambridge Health Alliance, Emory University, Northwestern University, and Duke University). Each patient was diagnosed with either bipolar disorder type II or major depressive disorder (MDD) and also met DSM-IV criteria for a MDE, while presenting 2 or 3 (but not more nor less) DSM-IV manic criteria. They did not meet DSM-IV criteria for a mixed or manic episode. Randomization was stratified by two factors: subtype of depressive disorder (bipolar type II vs. MDD), and presence or absence of rapid cycling. Research pharmacists generated the allocation sequence and assigned participants to their groups. No research procedures were performed without research staff first obtaining signed informed consent from each patient. Study visits occurred weekly for six weeks. Ziprasidone dosing began at 40 mg/day and was increased by 20–40 mg weekly based on target symptoms and tolerability with a target range of 80–160 mg/day of ziprasidone.

Baseline psychotropic drugs were unchanged throughout the study.

Interviews and rating scales used included the Structured Clinical Interview for DSM-IV Axis I Disorders-Patient Edition (SCID) [10], the Montgomery Åsberg Depression Rating Scale (MADRS) [11], the Mania Rating Scale from SADS-C (MRS) [12], the Clinical Global Impression for Bipolar Disorder (CGI-BP) [13], Global Assessment of Functioning (GAF) [14], Systematic Assessment for Treatment Emergent Events (SAFTEE), the Barnes Akathisia Scale (BAS) [15], and the Simpson-Angus Scale (SAS) [16].

Laboratory tests, consisting of complete blood count (CBC) with differential, biochemistry profile, ECG and pregnancy test, were conducted prior to the acute phase and at study termination. Physical examination and vital signs were assessed at study screening and termination visits. Patient termination occurred if the patient experienced a worsening of MADRS scores greater than 30% above the baseline score in two successive visits, if MRS >20 in two successive visits, if suicidal ideation worsened as determined by a MADRS suicide item of ≥3 in two successive visits, or based on clinician judgment or patient preference.

Inclusion criteria for the study were as follows: male or female; aged 18–65 years; current DSM-IV diagnosis of bipolar disorder type II or MDD; currently meeting DSM-IV criteria for a MDE while presenting with 2 or 3 DSM-IV manic criteria; if female, nonpregnant/nonlactating; if sexually active using adequate contraception; not psychotic and no cognitive impairment. Specific mania criteria used are presented in the results. Minimum duration of the episode followed the standard DSM-IV definition of a MDE, namely 2 weeks or longer.

Exclusion criteria for the study were as follows: current substance abuse in the previous month or relapse to substance abuse during the study (as defined by meeting DSM-IV criteria); medically unstable as judged by the study investigators; lack of capacity to provide informed, written consent; previous intolerance to ziprasidone or current use of ziprasidone at study baseline or within 3 months of study entry; serious suicidality as evidenced by score of 3 or greater on suicide item of MADRS; previous diagnosed cardiac arrhythmias; current psychotic MDE; history of potentially lethal suicide attempt.

The primary endpoint measured MADRS scores over weeks in enrolled patients. Secondary measures tracked changes in CGI and MRS scores, a priori subgroup analyses based on monotherapy versus adjunctive therapy, and MDD versus bipolar type II diagnostic subtypes. Treatment response was defined as 50% improvement in MADRS and in MRS. Treatment remission was defined as MADRS ≤9 and YMRS ≤11.

Power analysis, with β = 0.20 and two-tailed α = 0.05, was based on pilot studies previously conducted for the mania registration trials which also included mixed episodes and assessed MADRS scores, with about ten point improvement with drug over placebo. Based on the available pilot data, a projected standard error of the mean difference was assumed to range from equivalent to the mean difference to twice as much as the mean difference (5–15 points), producing a projected sample size of about 100 patients. After about three years of recruitment, the study was terminated with a somewhat smaller overall sample after the recruitment period could not be extended.

All baseline measures between treatment arms were compared with endpoint measures. Effect sizes along with their 95% confidence intervals were reported. The proportions of subjects who improved reaching response/remission between the treatment arms were compared using chi-square and Fisher exact test statistics at endpoint. The main statistical analysis involved the use of a linear mixed effects repeated measures model. To account for the correlated nature of the data, we analyzed patients’ data as repeated measures (using unrestricted covariance structure) and included site of data collection as a random effect. Backward selection procedure was used to select important predictors. The primary model was built using MADRS scores over time as the response variable, ziprasidone (drug) as the main explanatory variable, and weeks (time) and its interaction with drug arm as the dependent variables in the model.

A secondary exploratory analysis was performed with baseline measures (MADRS, race, diagnosis, and interaction between diagnosis and drug). Even though race and baseline severity were only marginally significant, they were forced into the model due to the significant differences in the racial composition of the two treatment arm groups. When analyzed with adjustment for race and baseline severity of depression, the overall results did not differ notably. Model assumptions were verified. Sensitivity analysis assessing robustness of results excluding one outlier data point was done. The only notable difference from the original results was the change from marginality (p = 0.104) to significance (p = 0.023) in the difference between temporal patterns in the MADRS scores for the two treatment arms. To address this issue we analyzed the data slices separately for each week and found that the treatment effect was significant at weeks 3, 5, and 6, and marginally significant at week 4. Analyses were completed in Stata 11 (StataCorp LP, College Station, TX), and SAS 9.2 (SAS Institute Inc., Cary, NC).

Dropout rates were not significantly different overall (attrition was 19% of the ziprasidone group versus 21% of the placebo group) and over time of the study; for dropouts by randomization arm and CONSORT data see Figure S1.

In the two a priori secondary subgroup analyses, we assessed treatment response by diagnostic subtype, and by concomitant medications used (comparing those taking mood stabilizer or antidepressant versus not). Since this study was powered for the primary outcome only, we kept this marginally significant interaction in the model and assessed these secondary moderators of outcome through separate descriptive stratified analyses, with mean differences or relative risks and confidence intervals.

## Results

Clinical and demographic characteristics of the sample are provided in Table 1. Specific mania criteria identified were as follows, in descending order of frequency: flight of ideas (59%), distractibility (58%), decreased need for sleep (43%), impulsive behavior (27%), pressured speech/increased talkativeness (24%), increased goal-directed activities (22%), and grandiosity (15%). Thus, the most common presentation was an acute MDE with flight of ideas, distractibility, and decreased need for sleep.

**Table 1:** Clinical and Demographic Characteristics of the Sample.

	Ziprasidone (n = 35)	Placebo (n = 38)
Age (mean±SD, years)	39.1±11.9	38.7±12.7
Gender, % (n)		
Male	47.1 (16)	47.4 (18)
Female	52.9 (18)	52.6 (20)
Race, % (n)		
Non-caucasian	73.5 (25)	44.7 (17)
Caucasian	26.5 (9)	55.3 (21)
Education Level, % (n)		
High School	60.0 (18)	59.4 (19)
Some College	30.0 (9)	18.8 (6)
Undergradute Degree	10.0 (3)	12.5 (4)
Graduate Degree	0 (0)	9.4 (3)
Diagnosis, % (n)		
MDD	44.1 (15)	36.8 (14)
BD Type II	55.9 (19)	63.2 (24)
Concurrent Medications, % (n)		
None	55.9 (20)	44.7 (17)
Antidepressants	35.3 (11)	44.7 (17)
Mood Stabilizers	0.0 (0)	7.9 (3)
Antidepressants & MoodStabilizers	8.8 (3)	2.6 (1)
Rapid Cycling, % (n)		
Non-rapid cyclers	80.0 (24)	74.2 (23)
Rapid cyclers	20.0 (6)	25.8 (8)
Past Substance Abuse, % (n)		
None	43.3 (13)	56.3 (18)
Positive History	56.7 (17)	43.3 (13)

SD = standard deviation, MDD = major depressive disorder, BD = bipolar disorder.

**Figure 1:**
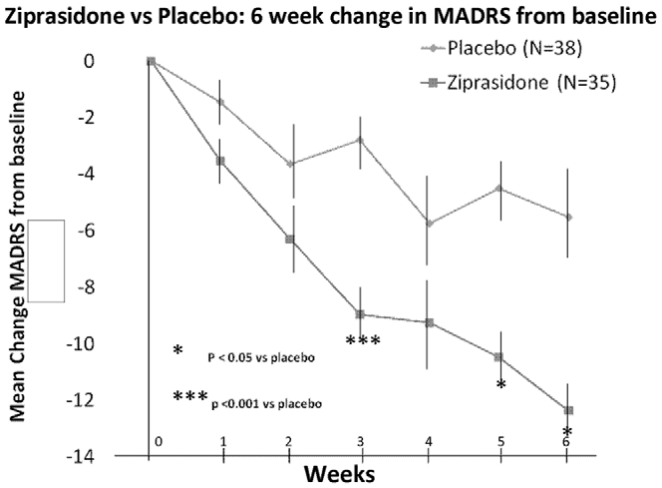
Ziprasidone vs Placebo: 6 week change in MADRS (SD) from baseline.

**Figure 2:**
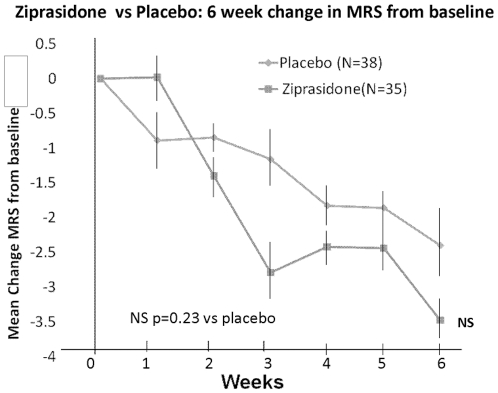
Ziprasidone vs Placebo: 6 week change in MRS (SD) from baseline.

As seen in Table 2 and Figure 1, there was a very significant effect of ziprasidone (p = 0.0038), weeks from baseline (p<0.001), and week*drug interaction (p = 0.0447). Table 3 shows raw outcome data from baseline to endpoint for the various rating scales. Treatment response by categorical group was 52.9% for ziprasidone versus 28.9% for placebo (χ^2^ = 4.29, df = 1, p = 0.04). Treatment remission by categorical group was 50.0% for ziprasidone versus 18.4% for placebo (χ^2^ = 8.05, df = 1, p = 0.0045). MRS scores did not change appreciably over time in both groups (Table 3, Figure 2).

**Table 2:** Final ANOVA table of model without baseline adjustments.

Effect	Estimate (β)	DF	DF	F Value	p-Value
Drug(Ziprasidone)	−1.53	1	419	8.49	0.0038
Week	−4.66	6	419	11.85	<.0001
Week*drug	−0.88	6	419	2.17	0.0447

DF =  degrees of freedom, ANOVA = Analysis of variance, β = effect estimate.

**Table 3:** Clinical Response of the Sample.

Outcome	Ziprasidone (n = 35)		Placebo (n = 38)		Ziprasidone vs. Placebo
	Baseline,mean ±SD	Endpoint,mean ±SD	Baseline,mean ±SD	Endpoint,mean ±SD	Outcome Change†Difference [95% CI]
MADRS	23.4±6.5	12.0±10.9	25.1±7.9	19.2±9.3	5.4 [0.6, 10.2]* ‡
MRS	8.4±6.1	4.7±5.2	8.8±6.2	6.5±5.1	1.5 [−1.1, 4.0]
CGI	4.0±0.9	2.8±1.2	4.1±0.9	3.5±1.1	0.5 [−0.1, 1.1]
GAF	56.7±5.9	65.8±9.3	56.2±5.4	60.9±7.9	4.4 [0.2, 8.6]*

SD = Standard deviation, MADRS = Montgomery-Åsberg Depression Rating Scale, MRS = Mania Rating Scale, CGI = Clinical Global Impressions Scale, GAF = Global Assessment of Functioning Scale.

* p<0.05.

† Outcome change = Change of each measure from baseline to endpoint.

‡ F = 8.273.

In secondary exploratory analyses (Appendix S1, Appendix S2 and Appendix S3), it was observed that while baseline MADRS was not notably different between drug and placebo groups, it was strong in predicting further MADRS scores (p<0.0001). MADRS scores exhibited significant changes over time in both groups (p<0.001), as well as marginally differential temporal changes between drug and placebo groups (p = 0.104, which became p = 0.022 when outlier observation was excluded). The effect of ziprasidone on MADRS scores was significantly different in the two diagnoses subtypes (p = 0.036), with more benefit in type II bipolar disorder than MDD. Appendix S3 provides the week by week effect estimates for the adjusted results in the mixed effects regression model.

When tolerability to the regimen was investigated, there was no significant weight change from baseline to endpoint in the ziprasidone versus the placebo group (0.5 vs. 0.6 lbs). Akathisia rates (defined as BAS scores >2) did not increase (ziprasidone, initial = 8.8%, endpoint = 11.8%; placebo, initial = 21.0%, endpoint = 13.2%). Other extrapyramidal symptom rates (defined as SAS >2) also did not increase (ziprasidone, initial = 5.8%, endpoint = 3.0%; placebo, initial = 10.8%, endpoint = 13.16%). Side effects reported by 5% or more subjects were headache (n = 7) and drowsiness (n = 6) in the drug group and headache (n = 5) and drowsiness (n = 5) in the placebo group. Side effects were reported in 54.8% of the ziprasidone group and 56.3% of the placebo group. Eleven (15.2%) patients terminated from the study, 6 for side effects (akathisia, sedation, migraines, chest pain, n = 1 each, n = 2 nonspecific) and 5 for other reasons (lack of efficacy n = 2; withdrawal of consent, noncompliance with appointments, and legal arrest unrelated to ziprasidone, n = 1 each). Average dose did not differ significantly between ziprasidone (129.7 mg) and placebo (126.1 mg) groups.

## Discussion

Ziprasidone was effective and relatively tolerable in this first randomized clinical trial of any medication for the acute depressive mixed state. These results provide preliminary support for both the nosological validity and the practical utility of a broadening of the concept of mixed mood episodes beyond current DSM constraints. Our definition of a depressive mixed state is provisional, and outside the DSM-IV framework. In secondary analyses, ziprasidone appeared somewhat more consistently effective in bipolar disorder type II than in MDD, but no notable difference was seen between monotherapy versus adjunctive use with antidepressants or mood stabilizers.

The clinical interpretation of the statistical results of the mixed effects regression model involve the following: Patients improved in both ziprasidone and placebo groups over the course of the six weeks of follow-up. Improvement with placebo likely reflects natural history of bipolar disorder, namely, that all episodes eventually end spontaneously, with depressive episodes usually lasting 3–6 months on average [17]. Given that these patients had been depressed for at least two weeks before entry into the study, some would recover spontaneously over the course of the next one to two months during the study. This clinical reality explains the statistical result of an effect of weeks from baseline. The week by drug interaction effect reflects the fact that this natural recovery was enhanced by treatment with ziprasidone, as opposed to no active treatment (placebo reflecting natural history alone). The independent effect of ziprasidone, irrespective of duration of follow-up, is also reflected in the statistical finding of a significant effect of the drug in the mixed effect regression model. In sum, patients recover gradually over time, but this recovery is enhanced by treatment with ziprasidone.

There are no previous randomized studies assessing neuroleptic response in the depressive mixed state, but studies have previously shown benefit with neuroleptics in dysphoric mania (DSM-defined mania with subsyndromal depressive symptoms). Perhaps any type of mixed state, whether predominantly depressive or predominantly manic, will be more responsive to neuroleptics [8]. It could also be that ziprasidone may be more preferentially effective in the depressive mixed state, due to its biochemical properties, consisting of high serotonin and norepinephrine reuptake properties with high 5HT-1A partial agonism (similar to what is seen with some antidepressant classes) [18]. This possibility is interesting especially given the inefficacy of ziprasidone in three studies of DSM-IV defined bipolar depression which includes pure depressive states (without any manic symptoms) and depressive mixed states (depression with some manic symptoms). In two monotherapy, similarly designed, randomized, double-blind, placebo- controlled six-week trials of ziprasidone (dose 40–160 mg/day) that included 900 subjects with adult bipolar I depression assigned to treatment [19], ziprasidone (Study 1 mean dose in low dose group = 54 mg/day, n = 540; mean dose in high dose group = 113 mg/day; Study 2 mean dose = 84 mg/day, n = 360) failed to separate from placebo on the primary outcome measure of change in total MADRS score from baseline to week 6 in mixed model repeated measures analyses of intent-to-treat populations. Secondary analyses showed that in both studies response rates (≥50% improvement from baseline MADRS scores) were similar in the ziprasidone and placebo groups (in study 1∶53% for ziprasidone 40–80 mg/d vs 46% for ziprasidone 80–160 mg/d vs 49% for placebo; in study 2∶53% vs 51% for ziprasidone vs. placebo respectively). A third adjunctive study (in which ziprasidone, 40–160 mg/d, was added to the mood stabilizers lithium, valproate or lamotrigine), was also a randomized, placebo-controlled, six-week trial. In this study of 298 randomized subjects, there were no significant differences between ziprasidone (mean dose = 89 mg/day) and placebo on the primary outcome measure of change in total MADRS score from baseline to week 6 (mean±standard error −13.24±1.24 vs −12.88±1.08 for ziprasidone vs placebo respectively, p = .792) and on key secondary outcome of change from baseline to week 6 in CGI-S scores (mean±standard error −1.04±0.2 for both groups, p = .722). There was no difference in efficacy across groups when subdivided by mood stabilizer. These monotherapy data have been reported in research conferences [20], and the adjunctive study has been published [19]. Dosing may also be relevant: in these negative studies, the mean ziprasidone dose was 89.8±29.1 mg/day, whereas in our study, the dose was higher (129.7±45.3 mg/day).

Previous studies have suggested that DSM-defined MDEs involve the depressive mixed state (presence of manic symptoms) in 25–50% of cases [21], [22]. If this is so, then perhaps this study, combined with the other studies showing lack of benefit in DSM-defined bipolar depression, demonstrates that ziprasidone may be preferentially effective in the depressive mixed state, but not in pure bipolar depression. The lack of benefit seen in the DSM-defined bipolar depression studies may thus have been due to a watering down of the benefit in the 25–50% of subjects with depressive mixed states by lack of benefit in the 50–75% of subjects with pure depressive states.

The clinical implications of this possibility include the diagnostic importance of assessing all depressed patients for current manic symptoms, irrespective of whether or not they are diagnosable with bipolar disorder in the past, and irrespective of whether or not concurrent manic symptoms meet DSM-defined thresholds for mania or hypomania [6], [7]. Thus, depression with flight of ideas and brief periods of hyperactivity (decreased need for sleep) would likely be responsive to ziprasidone, whereas pure melancholic depression without any flight of ideas and with constant low energy would be less responsive. It may be, based on our secondary outcomes, that there are still differences between mixed depression in MDD versus bipolar disorder type II.

All studies have limitations, and in this case one potential limitation in relation to secondary analyses would be sample size. The randomized study design should account for most potential confounding effects, but residual confounding cannot be completely eliminated without larger studies. Additionally, sufficient assessment of the integrity of double blind procedure was not completed. Replication of these results is also needed to confirm validity and generalizability in other samples.

### Conclusion

We observed a statistically significant difference between ziprasidone and placebo, especially in type II bipolar disorder, in this first randomized study of any medication for the depressive mixed state. These results provide preliminary support for both the nosological validity and the practical utility of a broadening of the concept of mixed mood episodes. Further research will need to clarify the specific nature of such potential mixed mood states, as well as their treatment.

## Supporting Information

Checklist S1 CONSORT Checklist.
(DOC)

Protocol S1 Trial Protocol.
(DOC)

Figure S1 CONSORT Flowchart.
(TIF)

Appendix S1 ANOVA table of model with baseline adjustment.
(DOC)

Appendix S2 Baseline adjusted fixed effects model table.
(DOC)

Appendix S3 Week by week table of mixed effects regression model.
(DOCX)
