# Supplementary material for: A 6 Week Randomized Double-Blind Placebo-Controlled Trial of Ziprasidone for the Acute Depressive Mixed State
Source: PLoS One. 2012 Apr 24;7(4):e34757. doi: 10.1371/journal.pone.0034757 (PMC3335844; doi:10.1371/journal.pone.0034757)
Supplement: Protocol S1 — Trial Protocol. (DOC) [file pone.0034757.s002.doc]

# **Double-Blind Placebo-Controlled Trial of Ziprasidone (Geodon®)**

# **for the Depressive Mixed State**

**Co-PIs**: S. Nassir Ghaemi MD MPH, Prakash Masand MD, Ashwin A. Patkar MD MRCPsych, William S. Gilmer MD

Sites: Mood Disorders Program, Tufts Medical Center; Mood Disorders Research Program, Duke University Medical Center; Clinical Trials Program, Northwestern University (Chicago)

Correspondence to:

S. Nassir Ghaemi MD MPH, Tufts Medical Center 800 Washington Street, #1007, Boston, MA 02111. Phone: 617-636-5735, Fax: 617-636-4852, email: nghaemi@tuftsmedicalcenter.org

Contact: Elizabeth Whitham

Research Study Coordinator

Mood Disorders Program

Tufts Medical Center

800 Washington Street, #1007

Boston, MA 02111

Phone: 617-636-3025

Fax: 617-636-4852

TABLE OF CONTENTS

I. BACKGROUND AND RATIONALE

II. STUDY OBJECTIVES

III. STUDY DESIGN

IV. STUDY POPULATION

1. Number of Subjects
2. Subject Recruitment

C. Inclusion Criteria

D. Exclusion Criteria

V. STUDY PROCEDURES

A. Blinding and Controls

B. Dosage Regimen

C. Visit-Specific Treatment and Evaluation Sequence

D. Concomitant Medication and Therapy

VI. STUDY MEDICATION

A. Description and Dispensing

B. Storage

VII. CLINICAL ASSESSMENTS

VIII. STATISTICAL EVALUATION

1. Power Analysis
2. Randomization Method
3. Outcome Measures and Planned Analyses
4. Definition of Evaluable Subject (safety and efficacy)
5. RISKS AND DISCOMFORTS
6. POTENTIAL BENEFITS

XI. ETHICAL AND REGULATORY CONSIDERATIONS

A. Institutional Committee Approval

B. Informed Consent

C. Document Storage and Retention

D. Protocol Amendments

E. Confidentiality of Data and Subject Records

XII. ADDITIONAL INFORMATION REGARDING STUDY CONDUCT

A. Case Report and Other Forms

B. Quality Assurance Audits

C. Study Discontinuation/Termination

1. Publication or Presentation of Results
2. STUDY DESIGN
3. STUDY VISIT SCHEDULE
4. REFERENCES
5. **BACKGROUND AND RATIONALE**

Mixed states in bipolar disorder have long been recognized. Over a century ago, Weygandt argued that mixed states were the most common presentations in manic-depressive illness [1], and later Kraepelin agreed [2]. While Kraepelin identified pure depression and pure mania, he described 6 different mixed states, which he considered more prevalent than pure mood states [2].

In DSM-IV, the definition of a mixed episode was narrowed, compared to previous definitions, so as to require full criteria for both mania and depression. This was not primarily based on empirical grounds, but rather, according to some members of the DSM-IV committee [3], so as to compensate for expansion of the bipolar diagnosis in other areas (eg., inclusion of type II and rapid cycling definitions).

In the last decade, a number of studies have suggested that the previous broader definitions of mixed states may have diagnostic validity and therapeutic utility. Empirical studies support the possible validity of definitions of mixed state in which manic episode criteria are met with two or more depressive criteria (dysphoric mania) [4]. Using this definition, for instance, one study demonstrated greater treatment response with valproate than with lithium [5]. Other clinical studies suggest that a mixed state may be present when major depressive episode criteria are met with at least one manic criterion (such as racing thoughts) [6, 7]. In those clinical studies, such mixed states appeared less responsive to antidepressants and more responsive to adjunctive mood stabilizers [8].

Using the narrow DSM-IV criteria, less than 10% of episodes in patients with bipolar disorder would meet criteria for a mixed episode. Using broader mixed states criteria incorporating the clinical pictures of dysphoric mania and agitated depression, similar data suggest that about 50% of episodes in bipolar disorder would be diagnosable as mixed states. One study found that only 54% of 143 broadly defined mixed states (using Kraepelinian definitions) met DSM-III-R criteria for the mixed episode [9].

It should be noted that this study of the depressive mixed state differs from a study of “agitated depression”. The concept of agitated depression does not have a consensus definition; usually it entails a major depressive episode with at least psychomotor agitation. The depressive mixed state, on the other hand, is defined as more than simply psychomotor agitation along with depression: there must also be one or two other manic symptoms (depending on the definition). Three more manic symptoms would define the condition as a DSM-IV mixed episode. Thus the depressive mixed state is a subthreshold DSM-IV mixed episode.

Studies suggest that the majority of persons with a depressive mixed state have bipolar disorder type II, but a substantial group will also have unipolar depression, according to DSM-IV criteria [7].

Ziprasidone appeared to be especially effective in treating manic patients who met DSM-IV criteria for a mixed episode in its registration trials. Due to unequal sample sizes in randomized groups, however, only 4 patients were randomized to placebo among those with mixed episodes (Pfizer, data on file). Nonetheless, those pilot data, along with the biochemical effects of ziprasidone on serotonergic neurotransmission, support the potentially selective utility of this agent in those with a mixture of manic and depressive symptoms.

The purpose of the proposed study is to examine the efficacy of ziprasidone for the depressive mixed state in patients with bipolar or unipolar depression. This will be the first double-blind RCT in the depressive mixed state. Pfizer is sponsoring this study.

.

## STUDY OBJECTIVES

The primary outcome of this study is to determine if ziprasidone is effective for the depressive mixed state in bipolar and unipolar depression.

1. **STUDY DESIGN**

This will be a four-site, block randomized (1:1 ratio) double-blind, placebo-controlled prospective study in which 100 patients will be enrolled. Patients will be randomized to ziprasidone or placebo in the 6 week acute phase of the study. Randomization will be stratified by three factors: subtype of depressive disorder (bipolar type II vs. unipolar), presence or absence of rapid cycling and duration of current MDE (< vs. > 3 months). The primary outcome measure will be change from baseline Montgomery-Asberg Depression Rating Scale scores in the acute phase. Enrollment will occur over 12 months.

###### STUDY POPULATION

#### Number of Subjects

We plan on enrolling 100 subjects overall, 25 subjects at the Tufts Medical Center site. A full power analysis is provided in section VIIIA.

#### Subject Recruitment

In order to avoid any appearance of coercion by the treating clinician, study coordinator Elizabeth Whitham will always conduct initial presentation of the study.

#### Inclusion Criteria

1. Age 18-65
2. Diagnosis of Bipolar Disorder Type II, or unipolar major depressive disorder
3. If female, nonpregnant/nonlactating
4. If a sexually active female of reproductive potential, must be using adequate contraception (i.e., oral contraceptives, barrier protection, or prior tubal ligation)
5. Currently meets DSM-IV criteria for a major depressive episode, and presence of 2 or 3 DSM-IV manic criteria
6. All other baseline psychotropic drugs will be allowed to be continued unchanged. However, if antidepressant use had been initiated in the previous 2 months, or was thought to be contributing to the depressive mixed state, then antidepressant medications would be discontinued 2 weeks (5 weeks for fluoxetine) before initiating the study.
7. Decisional capacity will be assessed by Dr. Ghaemi utilizing the standard psychiatric approach to assessing capacity:

- Factual understanding
- Appreciation of available options
- Rational thought process
- Communication of awareness

Please note that the subjects invited to participate are not psychotic, and do not have cognitive impairment.

#### Exclusion Criteria

1. Substance abuse or dependence in the previous month or positive urine drug screen at screening or relapse during study to substance abuse (meeting DSM-IV criteria).
2. Medically unstable as judged by study investigators
3. Lack of capacity to provide informed, written, consent to investigators
4. Previous intolerance to ziprasidone or current use of ziprasidone at study baseline or within 3 months of study entry.
5. Serious suicidality as evidenced by score of 3 or greater on suicide item of MADRS
6. Previous diagnosed cardiac arrhythmias
7. Current psychotic major depressive episode
8. History of potentially lethal suicide attempt

##### STUDY PROCEDURES

### Blinding and Controls

The study will be double blind and randomized. There will be two arms, ziprasidone and placebo, that will serve as comparators. All subjects will be offered the option of an open label courtesy trial of up to 8 weeks of treatment with ziprasidone at no charge upon their study conclusion. Subjects who do not complete the full 6-week double-blind trial will be eligible for the open courtesy trial after the baseline visit, given that they have been found eligible based on the inclusion and exclusion criteria.

### Dosage Regimen

The once-daily total daily dose will be 80-160 mg/d of ziprasidone. Dosing will begin at 20 mg BID with a flexible dose escalation strategy based on target symptoms and tolerability, with a target dose range of 80-160 mg/d. Dose escalations will occur by increments of 20-40 mg weekly.

### Visit-Specific Treatment and Evaluation Sequence

Visits will be planned to occur at the screening visit and weeks 0, 1, 2, 3, 4, 5, 6. Research scales will be administered at each visit. For a detailed description of which scales are administered per visit, please see “XIV. Study Visit Schedule” on page 21. No research procedures will be performed without a patient first signing an informed consent. Laboratory tests, consisting of CBC with differential, biochemistry profile, ECG and urine pregnancy test, will be conducted prior to the acute phase, prior to the continuation phase, and at study termination. Physical examination and vital signs will be assessed at study entry and termination visits. Patient termination will occur if the patient experiences a worsening of MADRS scores greater than 30% above the baseline score in two successive visits or if MRS > 20 in two successive visits, or based on clinician judgment or patient preference.

1. Concomitant Medication and Therapy

All allowed concomitant medications will be unchanged throughout the study.

###### STUDY MEDICATION

1. Description and Dispensing

All medications will be dispensed by licensed research pharmacies.

1. Storage

All study medications at the Cambridge Health Alliance site will be stored in a double-locked cabinet in the main hospital pharmacy offices, accessible only to the study investigator and dispensed by staff pharmacists.

###### CLINICAL ASSESSMENTS

1. Structured Clinical Interview for DSM-IV Axis I Disorders-Patient Edition (SCID) [10] - The SCID mood module for current major depressive and manic syndromes will be administered at screening to verify DSM-IV diagnosis
2. Montgomery Asberg Depression Rating Scale (MADRS) [11] - The MADRS is a standard antidepressant rating scale, which is most sensitive to core mood symptoms in bipolar depression.
3. Quick Inventory for Depressive Symptoms (QIDS) [12] - A16-item clinician rated depression scale similar to the MADRS but which focuses on atypical symptoms.
4. Mania Rating Scale from SADS-C (MRS) [13]- This is derived from the SADS and has been used in numerous clinical trials of mania
5. Hamilton Scale of Anxiety (HAM-A) [14]- 12 item assessment on a scale of 0-4 of anxious mood; mostly dealing with physical symptoms.
6. Quality of Life Enjoyment and Satisfaction Questionnaire (Q-LES-Q) [15]- A weekly comprehensive self-rated quality of life questionnaire which targets specific areas of physical health/activities, feelings, work, household duties, school/course work, leisure activities, social relations and general activities.
7. Scale to Assess Unawareness of Mental Disorder (SUMD) [16]–This is a validated reliable scale used in the DSM-IV field trials to assess insight (awareness of illness). Insight may improve with ziprasidone, which would be an important predictor of compliance and improved long-term clinical prognosis.
8. Drug Attitude Inventory (DAI) [17]– This is a patient self report of subjective attitude toward taking medications, and again correlates with compliance.
9. Clinical Global Impression for Bipolar Disorder (CGI-BP) [18]– This is also an NIMH-derived scale that allows for separate global assessments of depressive, manic, and prophylactic efficacy in bipolar disorder.
10. Global Assessment of Functioning (GAF) [19]– The GAF is a single item clinician-rated scale that indicates overall level of psychosocial function during a specific period.
11. Longitudinal Internal Follow-up Evaluation: Range of Impairment of Functioning Tool (LIFE-RIFT) [20]- A scale to assess social/occupational functioning.
12. Systematic Assessment for Treatment Emergent Events – General Inquiry
13. Barnes Akathisia Scale (BAS) [21]- A 4-item clinician rated scale to assess symptoms of akathisia.
14. Simpson-Angus Scale for extrapyramidal symptoms (SAS) - A 10-item clinician rated scale to assess the presence and severity of extrapyramidal symptoms.
15. The structured Family History Screen (FHS) [22]- A brief family history in a structured manner to collect lifetime family psychiatric history and verify subject symptoms and treatment, to be completed by the subject.
16. The Temperament and Affective Disorders Questionnaire (TEMPS-A) [23]- 110-item self-rated true or false questionnaire to assess temperament.
17. Past Medication Response Structured Assessment - A structured history of past response to medications to be administered during screening.
18. PTSD Scale - 10 item scale created by Co-Principal Investigator, Dr. Gilmer, to assess past history of trauma, to be administered during screening.

###### STATISTICAL EVALUATION

1. Power Analysis

A power analysis was conducted. With a goal for β=0.20, and two-tailed α=0.05, the pilot studies previously conducted for the mania registration trials suggested that a mean change in MADRS scores were 20 points improved with ziprasidone compared to placebo. Given the few patients in the placebo arm, we conducted this power analysis with a smaller expected effect size, using a high projected mean difference of 10 points, and a low projected mean difference of 5 points. Based on the available pilot data, a projected standard error of the mean difference was assumed to be range from equivalent to the mean difference to twice as much as the mean difference (5-15 points). Using an internet-based power analysis software program, we conducted the power analysis noted in the table.

To assess various levels of effect size, power, and standard deviation, a sensitivity analysis was conducted (Table). As indicated by the table, if the mean difference is small and the standard deviation is large, then very large samples would be needed. However, if the same or larger effect was seen with somewhat larger standard deviation, the total sample size required would range from 26 to 138 patients. Thus, unless the true mean difference is quite minimal, a sample size in that range should have a good chance of detecting a real effect.

Table. Sensitivity analysis for sample size calculation

| Sample size (per arm) | Effect size (Mean difference in MADRS) | Standard deviation within each group | Power |
| --- | --- | --- | --- |
| 13 | 5 | 5 | .80 |
| 50 | 5 | 10 | .80 |
| 69 | 5 | 10 | .90 |
| 112 | 5 | 15 | .80 |
| 30 | 10 | 5 | .90 |
| 13 | 10 | 10 | .80 |
| 18 | 10 | 10 | .90 |
| 29 | 10 | 15 | .80 |
| 5 | 20 | 10 | .90 |

Therefore, a sample size of about 100 patients should be optimal for this study. This would be best accomplished in 4 sites, with a goal of accrual of 25 patients per site. At an average recruitment of about 2 patients per month, this could be achieved in one year of recruitment.

### Randomization Method

Randomization will occur with randomly generated computer lists held at the Duke site. All patients will be stratified by the following characteristics in block randomization:

Subtype of depressive disorder (bipolar type II vs. unipolar), presence or absence of rapid cycling, and duration of current MDE (< vs. > 3 months). These features may be unequally distributed in a small (<50) randomized study and could lead to high placebo response if type II, rapid cycling, or short duration episodes are overrepresented in the placebo group. Stratification will ensure control of these factors.

1. Outcome Measures and Planned Analyses

The a priori primary outcome measure will be change from baseline in MADRS scores in the acute phase. A priori secondary measures will be change from baseline in HAM-A, MRS, and other scores. The primary analysis for the acute phase between group effect size (for ziprasidone vs. placebo) will be obtained by unpaired t-test comparison and by mixed effects regression modeling. A priori subgroup analysis in rapid cyclers versus nonrapid cyclers, unipolar vs. bipolar subtypes, and shorter versus longer duration episodes, is also planned.

1. Definition of Evaluable Subject (Safety and Efficacy)

Subjects will be defined as able to be evaluated by two methods. The primary method will be an intent-to-treat analysis, including those who discontinue drug due to lack of efficacy or due to side effects. The secondary method will be completer analysis.

###### RISKS AND DISCOMFORTS

The Most common risks associated with use of ziprasidone that occurred in > 30% of exposed patients, based on placebo-controlled trials, include: somnolence, tremor or involuntary muscle movement. Other risks that occurred in between 5% and 16% of patients receiving ziprasidone were restlessness, nausea, vomiting, rash, and dizziness. During clinical trials, a small percentage (0.4%) of patients experienced seizures with ziprasidone.

Pertinent laboratory monitoring during treatment with ziprasidone include complete blood counts (CBCs) and hepatic enzyme levels. Baseline biochemistry profiles also will be obtained to assure that hepatic enzyme levels are within normal range. Women of childbearing potential will have pregnancy tests at the screening and termination visits to assure they are not pregnant and must use a suitable form of contraception during their participation in the protocol.

Venipuncture in order to monitor serum valproate levels, as well as relevant laboratory parameters, may cause temporary bruising and discomfort to subjects. This risk will be minimized by the use of trained and experienced phlebotomists to perform venipuctures.

Subjects may become upset or feel uncomfortable with questions used in the clinical assessments. This risk will be minimized by allowing subjects to skip and go to the next question if they become uncomfortable.

There is an inherent and unavoidable risk for worsening of the underlying psychiatric condition (either depression or else cycling from depression to mania or mixed mania) due to the natural course of illness and possible lack of efficacy with either active drug or placebo. This risk will be minimized by close, careful and regular monitoring of psychiatric symptoms by research staff throughout the protocol. For subjects whose clinical conditions may worsen, the investigator may deem it appropriate to discontinue the study protocol and arrange for routine care outside of the research study.

###### POTENTIAL BENEFITS

Patients do not pay for the treatments they receive. They will receive reimbursement for travel and parking expenses.

All subjects will be offered the option of an open label courtesy trial of up to 8 weeks of treatment with ziprazidone at no charge upon their study conclusion. Subjects who do not complete the full 6-week double-blind trial will be eligible for free clinical open treatment with ziprazidone for at least 2 months if they complete an initial baseline visit.

###### ETHICAL AND REGULATORY CONSIDERATIONS

### Institutional Committee Approval

The Institutional Review Boards of the participating institutions will need to approve the protocol.

1. Informed Consent

Participation in this study is voluntary and at no cost to the subjects. All subjects to be entered into the study must be thoroughly informed of the details of the study. Prior to participation in this trial, each subject will have an opportunity to ask questions and will sign a written informed consent statement, which must be witnessed. The informed consent will be obtained by study the PI, Dr. Ghaemi. The signed consent forms will be filed with the investigator’s copies of the Case Report Forms for each subject. Any subject may voluntarily withdraw from the study at any time without prejudicing treatment.

### Document Storage and Retention

Documents from Tufts Medical Center will be stored at the offices of the Tufts Medical Center Mood Disorders Program at 800 Washington Street, Boston, MA 02111.

### Protocol Amendments

Any protocol amendments will be accompanied by IRB approval and appropriate revisions of consent form documents.

### Confidentiality of Data and Subject Records

Subject research charts will only be identifiable by initials and numbers. The forms used during the screening visit or subsequent visits which may contain substance abuse information will be coded and deidentified, and kept secure and locked in the offices of the Mood Disorders Program Principal Investigator and research coordinator. No subjects will be identifiable in any published data produced from this study.

###### ADDITIONAL INFORMATION REGARDING STUDY CONDUCT

### Case Report and Other Forms

All case report and other forms will be contained in the study research chart of each subject.

### Quality Assurance Audits

Charts will be made available as requested to the sponsor, the participating site IRBs, and the FDA or other federal regulatory bodies for quality assurance purposes.

1. Study Discontinuation/Termination

Patients will be discontinued from the study for the following reasons:

1. 30% worsening of MADRS compared to baseline on two successive visits
2. MRS > 20 in two successive visits
3. Worsening of suicidal ideation (SI) as determined by MADRS suicide item ≥ 3 in two successive visits.
4. Clinical worsening sufficient to require hospitalization or other medication interventions in the judgment of the treating psychiatrist
5. Patient noncompliance with medications or appointments
6. Patient preference

After study termination, subjects who do well will receive Geodon. Subjects who do not do well will have the option of receiving Geodon. The blind will be broken only if there is a medical emergency.

D. Serious Adverse Event Reporting

For those studies where the principal investigator is required to report serious adverse advents (SAEs) to Pfizer, the investigator must use the enclosed Serious Adverse Event Fax Cover Sheet together with the FDA’s MEDWATCH form FDA 3500A (for mandatory reporting) which can be downloaded at [www.FDA.gov](http://www.FDA.gov/).

Reporting of Serious Adverse Events: Within 24 hours of first awareness of the event (immediately if the event is fatal or life-threatening), Principal Investigator will report to Pfizer by facsimile any Serious Adverse Event (“SAE,” as defined below) that occurs during the SAE reporting period (as defined below) in a Study subject assigned to receive the Pfizer Product (see Section 5, Pfizer Product). Principal Investigator will report such SAEs using an FDA MEDWATCH form and the *Serious Adverse Event Fax Cover Sheet* provided by Pfizer. SAEs should be reported as soon as they are determined to meet the definition, even if complete information is not yet available. SAEs will be reported to the Tufts Medical Center IRB on the same schedule as the study sponsor.

SAE Definition: An SAE is any adverse event, without regard to causality, that is life-threatening or that results in any of the following outcomes: death; in-patient hospitalization or prolongation of existing hospitalization; persistent or significant disability or incapacity; or a congenital anomaly or birth defect. Any other medical event that, in the medical judgment of the Principal Investigator, may jeopardize the subject or may require medical or surgical intervention to prevent one of the outcomes listed above is also considered an SAE. A planned medical or surgical procedure is not, in itself, an SAE.

SAE Reporting Period: The SAEs that are subject to this reporting provision are those that occur from after the first dose of the Pfizer Product through 28 days after discontinuation of the Pfizer Product.

Follow-Up Information: Institution will assist Pfizer in investigating any SAE and will provide any follow-up information reasonably requested by Pfizer.

Regulatory Reporting: Reporting an SAE to Pfizer does not relieve Institution of responsibility for reporting it to the FDA, as required.

E. Publication and Presentation of Results

All data will be recorded, stored, and analyzed by the Duke and Tufts Medical Center sites. The co-PIs are solely responsible for ultimate presentation and publication of data produced by this study. The study enrollment and follow-up should be complete by the end of 2009. Study results will plan to be presented at the American Psychiatric Association meeting of 2009, and any other meetings as feasible. Publication of the main final manuscript will be planned for 2009. It is expected that two to three other manuscripts assessing secondary outcome measures will also be published by 2009.

###### STUDY DESIGN

**Placebo**

6 weeks

6 weeks

**Ziprasidone**

**Parallel step**

Stratify by: Subtype of bipolar disorder (type I vs. type II/NOS, Presence of rapid cycling, Duration of current MDE (< vs. > 3 months)

**T**

**e**

**rm**

**i**

**n**

**a**

**t**

**i**

**o**

**n**

**R**

**a**

**n**

**d**

**om**

**i**

**z**

**a**

**t**

**i**

**o**

**n**

###### STUDY VISIT SCHEDULE

|  | **Screening** | **Weeks** | | | | | | | | | | | | | | | |
| --- | --- | --- | --- | --- | --- | --- | --- | --- | --- | --- | --- | --- | --- | --- | --- | --- | --- |
|  |  | **0** | **1** | | | **2** | | | **3** | | | **4** | | | **5** | | **6** |
| **VISITS:** | | | | | | | | | | | | | | | | | |
| Office Visits | • | • | • | | | • | | | • | | | • | | | • | | • |
| **PSYCHOMETRIC SCALES:** | | | | | | | | | | | | | | | | | |
| Demographics Form | • |  | |  | | |  | | |  | | |  | |  | |  |
| SCID Depression & Mania Modules | • |  | |  | | |  | | |  | | |  | |  | |  |
| Montgomery Asberg Depression Rating Scale (MADRS) | • | • | | • | | | • | | | • | | | • | | • | | • |
| Quick Inventory for Depressive Symptoms (QIDS) | • | • | | • | | | • | | | • | | | • | | • | | • |
| Mania Rating Scale (MRS) | • | • | | • | | | • | | | • | | | • | | • | | • |
| Hamilton Scale of Anxiety (HAM-A) |  | • | | • | | | • | | | • | | | • | | • | | • |
| Quality of Life Enjoyment and Satisfaction Questionnaire (Q-LES-Q) |  | • | | • | | | • | | | • | | | • | | • | | • |
| Scale to Assess Unawareness of Mental Disorder (SUMD) |  | • | | • | | | • | | | • | | | • | | • | | • |
| Drug Attitude Inventory (DAI) |  | • | | • | | | • | | | • | | | • | | • | | • |
| Clinical Global Impression Bipolar Depression (CGI-BP) |  | • | | • | | | • | | | • | | | • | | • | | • |
| Global Assessment of Function (GAF) |  | • | | • | | | • | | | • | | | • | | • | | • |
| LIFE-RIFT |  | • | | • | | | • | | | • | | | • | | • | | • |
| Systematic Assessment for Treatment Emergent Events |  | • | | • | | | • | | | • | | | • | | • | | • |
| Barnes Akathisia Scale (BAS) |  | • | | • | | | • | | | • | | | • | | • | | • |
| Simpson-Angus Scale for extrapyramidal symptoms (SAS) |  | • | | • | | | • | | | • | | | • | | • | | • |
| The structured Family History Screen (FHS) |  | • | |  | | |  | | |  | | |  | |  | |  |
| The Temperament and Affective Disorders Questionnaire (TEMPS-A) |  | • | |  | | |  | | |  | | |  | |  | |  |
| Side Effects Checklist |  | • | | • | | | • | | | • | | | • | | • | | • |
| Past Medication Response Structured Assessment |  | • | |  | | |  | | |  | | |  | |  | |  |
| PTSD Scale |  | • | |  | | |  | | |  | | |  | |  | |  |
| **MEDICAL ASSESSMENTS:** | | | | | | | | | | | | | | | | | |
| PE & Vital Signs |  | • | | |  | | |  | | |  | | |  | |  | • |
| Safety Labs | • |  | | |  | | |  | | |  | | |  | |  | • |

###### REFERENCES

1. Salvatore, P., et al., *Weygandt's On the Mixed States of Manic-Depressive Insanity: a translation and commentary on its significance in the evolution of the concept of bipolar disorder.* Harv Rev Psychiatry, 2002. **10**(5): p. 255-75.

2. Trede, K., et al., *Manic-depressive illness: evolution in Kraepelin's Textbook, 1883-1926.* Harv Rev Psychiatry, 2005. **13**(3): p. 155-78.

3. Dunner, D.L., *Bipolar disorders in DSM-IV: impact of inclusion of rapid cycling as a course modifier.* Neuropsychopharmacology, 1998. **19**(3): p. 189-93.

4. McElroy, S.L., et al., *Clinical and research implications of the diagnosis of dysphoric or mixed mania or hypomania.* Am J Psychiatry, 1992. **149**: p. 1633-44.

5. Swann, A.C., *Depression, mania, and feeling bad: the role of dysphoria in mixed states.* Bipolar Disord, 2000. **2**(4): p. 325-7.

6. Akiskal, H.S. and F. Benazzi, *Validating Kraepelin's two types of depressive mixed states: "depression with flight of ideas" and "excited depression".* World J Biol Psychiatry, 2004. **5**(2): p. 107-13.

7. Benazzi, F., *Depressive mixed state: testing different definitions.* Psychiatry Clin Neurosci, 2001. **55**(6): p. 647-52.

8. Koukopoulos, A. and A. Koukopoulos, *Agitated depression as a mixed state and the problem of melancholia.* Psychiatr Clin North Am, 1999. **22**(3): p. 547-64.

9. Perugi, G., et al., *Clinical subtypes of bipolar mixed states: validating a broader European definition in 143 cases.* J Affect Disord, 1997. **43**(3): p. 169-80.

10. Spearing, M.K., et al., *Modification of the Clinical Global Impressions (CGI) scale for use in bipolar illness (BP): the CGI-BP.* Psychiatry Research, 1997. **73**: p. 159-171.

11. Benazzi, F., *DSM-III-R schizophreniform disorder with good prognostic features: a six-year follow-up.* Can J Psychiatry, 1998. **43**(2): p. 180-2.

12. Rush, A.J., et al., *The 16-Item Quick Inventory of Depressive Symptomatology (QIDS), clinician rating (QIDS-C), and self-report (QIDS-SR): a psychometric evaluation in patients with chronic major depression.* Biol Psychiatry, 2003. **54**(5): p. 573-83.

13. Young, R.C., et al., *A rating scale for mania: Reliability, validity, and sensitivity.* Br J Psychiatry, 1978. **133**: p. 429-435.

14. Maier, W., et al., *The Hamilton Anxiety Scale: reliability, validity and sensitivity to change in anxiety and depressive disorders.* J Affect Disord, 1988. **14**(1): p. 61-8.

15. Endicott, J., et al., *Quality of Life Enjoyment and Satisfaction Questionnaire: a new measure.* Psychopharmacol Bull, 1993. **29**(2): p. 321-6.

16. Fiss, N. and A.C. Chaves, *Translation, adaptation and reliability study of the Scale to Assess Unawareness of Mental Disorder--SUMD.* Rev Bras Psiquiatr, 2005. **27**(2): p. 143-5.

17. Dolder, C.R., et al., *Brief evaluation of medication influences and beliefs: development and testing of a brief scale for medication adherence.* J Clin Psychopharmacol, 2004. **24**(4): p. 404-9.

18. Meaden, P.M., R.E. Daniels, and J. Zajecka, *Construct validity of life chart functioning scales for use in naturalistic studies of bipolar disorder.* J Psychiatr Res, 2000. **34**(3): p. 187-92.

19. Jones, S.H., et al., *A brief mental health outcome scale-reliability and validity of the Global Assessment of Functioning (GAF).* Br J Psychiatry, 1995. **166**(5): p. 654-9.

20. Leon, A.C., et al., *The Range of Impaired Functioning Tool (LIFE-RIFT): a brief measure of functional impairment.* Psychol Med, 1999. **29**(4): p. 869-78.

21. Barnes, T.R., *The Barnes Akathisia Rating Scale--revisited.* J Psychopharmacol, 2003. **17**(4): p. 365-70.

22. Weissman, M.M., et al., *Brief screening for family psychiatric history.* Archives of General Psychiatry, 2000. **57**: p. 675-682.

23. Akiskal, H.S., et al., *TEMPS-A: validation of a short version of a self-rated instrument designed to measure variations in temperament.* J Affect Disord, 2005. **85**(1-2): p. 45-52.

24. Reinert, D.F. and J.P. Allen, *The Alcohol Use Disorders Identification Test (AUDIT): a review of recent research.* Alcohol Clin Exp Res, 2002. **26**(2): p. 272-9.
