# Supplementary material for: A 6 Week Randomized Double-Blind Placebo-Controlled Trial of Ziprasidone for the Acute Depressive Mixed State
Source: PLoS One. 2012 Apr 24;7(4):e34757. doi: 10.1371/journal.pone.0034757 (PMC3335844; doi:10.1371/journal.pone.0034757)
Supplement: Appendix S1 — ANOVA table of model with baseline adjustment. (DOC) [file pone.0034757.s004.doc]

Appendix S1. ANOVA table of model with baseline adjustment

| Effect | Degrees of Freedom | F-value | p-value |
| --- | --- | --- | --- |
| Baseline MADRS | 1 | 59.39 | <0.00001 |
| Drug (Ziprasidone versus placebo) | 1 | 6.82 | 0.009 |
| Diagnosis (Type II BD vs MDD) | 1 | 0.69 | 0.41 |
| Week | 5 | 7.14 | 0.0000026 |
| Drug * Diagnosis | 1 | 4.41 | 0.036 |
| Week*Drug | 5 | 1.84 | 0.104 |
| Race | 1 | 1.98 | 0.160 |

MADRS = Montgomery Asberg Depression Rating Scale, BD = bipolar disorder, MDD = major depressive disorder
