# Supplementary material for: A 6 Week Randomized Double-Blind Placebo-Controlled Trial of Ziprasidone for the Acute Depressive Mixed State
Source: PLoS One. 2012 Apr 24;7(4):e34757. doi: 10.1371/journal.pone.0034757 (PMC3335844; doi:10.1371/journal.pone.0034757)
Supplement: Appendix S2 — Baseline adjusted fixed effects model table. (DOC) [file pone.0034757.s005.doc]

Appendix S2. Baseline adjusted fixed effects model table

| Effect | Week | Drug | Diagnosis | Race | Estimate (β) | SE | Df | t | p-value |
| --- | --- | --- | --- | --- | --- | --- | --- | --- | --- |
| Intercept |  |  |  |  | 7.25 | 2.71 | 4 | 2.68 | 0.05 |
| BL-MADRS |  |  |  |  | 0.64 | 0.08 | 345 | 7.66 | <0.0001 |
| Drug |  | Placebo |  |  | 0.47 | 2.08 | 345 | 0.22 | 0.82 |
| Drug |  | Ziprasidone |  |  | 0 |  |  |  |  |
| Diagnosis |  |  | 1 |  | 0.92 | 1.73 | 345 | 0.53 | 0.60 |
| Diagnosis |  |  | 2 |  | 0 |  |  |  |  |
| Week | 1 |  |  |  | 0 |  |  |  |  |
| Week | 2 |  |  |  | -2.33 | 1.09 | 345 | -2.14 | 0.03 |
| Week | 3 |  |  |  | -1.87 | 1.06 | 345 | -1.77 | 0.08 |
| Week | 4 |  |  |  | -4.59 | 1.64 | 345 | -2.79 | 0.005 |
| Week | 5 |  |  |  | -3.53 | 1.66 | 345 | -2.13 | 0.03 |
| Week | 6 |  |  |  | -4.74 | 1.75 | 345 | -2.71 | 0.007 |
| Drug*Diagnosis |  | Placebo | 1 |  | -4.48 | 2.45 | 345 | -1.82 | 0.07 |
| Drug*Diagnosis |  | Placebo | 2 |  | 0 |  |  |  |  |
| Drug*Diagnosis |  | Ziprasidone | 1 |  | 0 |  |  |  |  |
| Drug*Diagnosis |  | Ziprasidone | 2 |  | 0 |  |  |  |  |
| Week*Drug | 1 | Placebo |  |  | 0 |  |  |  |  |
| Week*Drug | 1 | Ziprasidone |  |  | 0 |  |  |  |  |
| Week*Drug | 2 | Placebo |  |  | -0.23 | 1.60 | 345 | -0.15 | 0.88 |
| Week*Drug | 2 | Ziprasidone |  |  | 0 |  |  |  |  |
| Week*Drug | 3 | Placebo |  |  | -3.97 | 1.53 | 345 | -2.60 | 0.01 |
| Week*Drug | 3 | Ziprasidone |  |  | 0 |  |  |  |  |
| Week*Drug | 4 | Placebo |  |  | -2.32 | 2.39 | 345 | -0.97 | 0.33 |
| Week*Drug | 4 | Ziprasidone |  |  | 0 |  |  |  |  |
| Week*Drug | 5 | Placebo |  |  | -4.03 | 2.40 | 345 | -1.68 | 0.09 |
| Week*Drug | 5 | Ziprasidone |  |  | 0 |  |  |  |  |
| Week*Drug | 6 | Placebo |  |  | -4.56 | 2.53 | 345 | -1.80 | 0.07 |
| Week*Drug | 6 | Ziprasidone |  |  | 0 |  |  |  |  |
| Race |  |  |  | 1 | -1.75 | 1.33 | 345 | -1.31 | 0.19 |
| Race |  |  |  | 2 | 0 |  |  |  |  |

BL-MADRS = baseline MADRS, df = degrees of freedom, SE = standard error

Diagnosis: 1 = bipolar disorder, type II; 2 = major depressive disorder

Race: 1 = white; 2 = non-white
