# Supplementary material for: A 6 Week Randomized Double-Blind Placebo-Controlled Trial of Ziprasidone for the Acute Depressive Mixed State
Source: PLoS One. 2012 Apr 24;7(4):e34757. doi: 10.1371/journal.pone.0034757 (PMC3335844; doi:10.1371/journal.pone.0034757)
Supplement: Appendix S3 — Week by week table of mixed effects regression model. (DOCX) [file pone.0034757.s006.docx]

| Week | Effect | DF | F | p |
| --- | --- | --- | --- | --- |
| 1 | Baseline MADRS | 60 | 59.6 | <0.000001 |
| 1 | Drug | 60 | 0.58 | 0.45 |
| 1 | Diagnosis | 60 | 1.28 | 0.26 |
| 1 | Drug*Diagnosis | 60 | 2.37 | 0.13 |
| 1 | Race | 60 | 1.01 | 0.32 |
| 2 | Baseline MADRS | 54 | 20.8 | <0.000001 |
| 2 | Drug | 54 | 1.43 | 0.24 |
| 2 | Diagnosis | 54 | 0.64 | 0.42 |
| 2 | Drug*Diagnosis | 54 | 3.90 | 0.05 |
| 2 | Race | 54 | 1.08 | 0.30 |
| 3 | Baseline MADRS | 49 | 32.2 | <0.000001 |
| 3 | Drug | 49 | 13.3 | 0.00065 |
| 3 | Diagnosis | 49 | 1.95 | 0.17 |
| 3 | Drug*Diagnosis | 49 | 6.06 | 0.017 |
| 3 | Race | 49 | 0.91 | 0.34 |
| 4 | Baseline MADRS | 48 | 4.92 | 0.03 |
| 4 | Drug | 48 | 3.40 | 0.07 |
| 4 | Diagnosis | 48 | 0.74 | 0.39 |
| 4 | Drug*Diagnosis | 48 | 2.15 | 0.15 |
| 4 | Race | 48 | 0.15 | 0.70 |
| 5 | Baseline MADRS | 47 | 7.18 | 0.01 |
| 5 | Drug | 47 | 5.37 | 0.02 |
| 5 | Diagnosis | 47 | 0.185 | 0.67 |
| 5 | Drug*Diagnosis | 47 | 1.27 | 0.27 |
| 5 | Race | 47 | 0.05 | 0.81 |
| 6 | Baseline MADRS | 47 | 4.01 | 0.05 |
| 6 | Drug | 47 | 6.75 | 0.01 |
| 6 | Diagnosis | 47 | 0.28 | 0.60 |
| 6 | Drug*Diagnosis | 47 | 2.22 | 0.14 |
| 6 | Race | 47 | 0.19 | 0.66 |

Appendix S3. Week by week table of mixed effects regression model

MADRS = Montgomery Asberg Depression Rating Scale

DF = degrees of freedom

* = interaction
